# Exercise ventilatory response after COVID-19: comparison between ambulatory and hospitalized patients

**DOI:** 10.1152/ajplung.00142.2023

**Published:** 2023-10-24

**Authors:** Ivan Guerreiro, Aurélien Bringard, Mayssam Nehme, Idris Guessous, Lamyae Benzakour, Alix Juillet De Saint Lager-Lucas, Anna Taboni, Frédéric Lador

**Affiliations:** ^1^Division of Pneumology, Department of Medicine, Geneva University Hospitals, Geneva, Switzerland; ^2^Faculty of Medicine, University of Geneva, Geneva, Switzerland; ^3^Division of Primary Care Medicine, Geneva University Hospitals, Geneva, Switzerland; ^4^Liaison Psychiatry and Crisis Intervention Service, Department of Psychiatry, Geneva University Hospitals, Geneva, Switzerland; ^5^Division of Radiology, Department of Imaging and Medical Information Sciences, Geneva University Hospitals, Geneva, Switzerland

**Keywords:** cardiopulmonary exercise testing, dyspnea, hyperventilation syndrome, post-COVID-19, ventilatory response

## Abstract

Inefficient ventilatory response during cardiopulmonary exercise testing (CPET) has been suggested as a cause of post-COVID-19 dyspnea. It has been described in hospitalized patients (HOSP) with lung parenchymal sequelae but also after mild infection in ambulatory patients (AMBU). We hypothesize that AMBU and HOSP have different ventilatory responses to exercise, due to different etiologies. We analyzed CPET realized between July 2020 and May 2022 of patients with persisting respiratory symptoms 3 mo after COVID-19. Chest computed tomography (CT) scan, pulmonary function tests, quality of life, and respiratory questionnaires were collected. CPET data were specifically explored as a function of ventilation (V̇e) and time. Seventy-nine consecutive patients were included (42 AMBU and 37 HOSP, median: 54 [44–60] yr old, 57% female). Patients were hospitalized for a median of 20 [8–34] days, with pneumonia (41%) or acute respiratory distress syndrome (ARDS; 30%). Among HOSP, 12(32%) patients had abnormal values for spirometry and 18(51%) for carbon monoxide diffusing capacity (*P* < 0.001). CPET showed no differences between AMBU and HOSP in peak absolute O_2_ uptake (V̇o_2_) (1.59 [1.22–2.11] mL·min^−1^; *P* = 0.65). Tidal volume (VT) as a function of V̇e, was lower in AMBU than in HOSP (*P* < 0.01) toward the end of exercise. The slope of the V̇e-CO_2_ production was higher than normal in both groups (30.9 [26.1–34.3]; *P* = 0.96). In conclusion, the severity of COVID-19 did not influence the exercise capacity, but AMBU demonstrated a less efficient ventilatory response to exercise as compared with HOSP. CPET with exploration of data as a function of V̇e and throughout the exercise better unveil ventilatory inefficiency.

**NEW & NOTEWORTHY** We evaluated the exercise ventilatory response in patients with persisting dyspnea after severe acute respiratory syndrome-coronavirus-2 (SARS-CoV-2) infection. We found that despite similar peak power and peak absolute O_2_ uptake, tidal volume as a function of ventilation was lower in ambulatory than in hospitalized patients toward the end of exercise, reflecting ventilatory inefficiency. We call for evaluation of minute ventilation with the exploration of data throughout the exercise and not only peak data to better unveil ventilatory inefficiency.

## Graphical abstract

**Figure:**
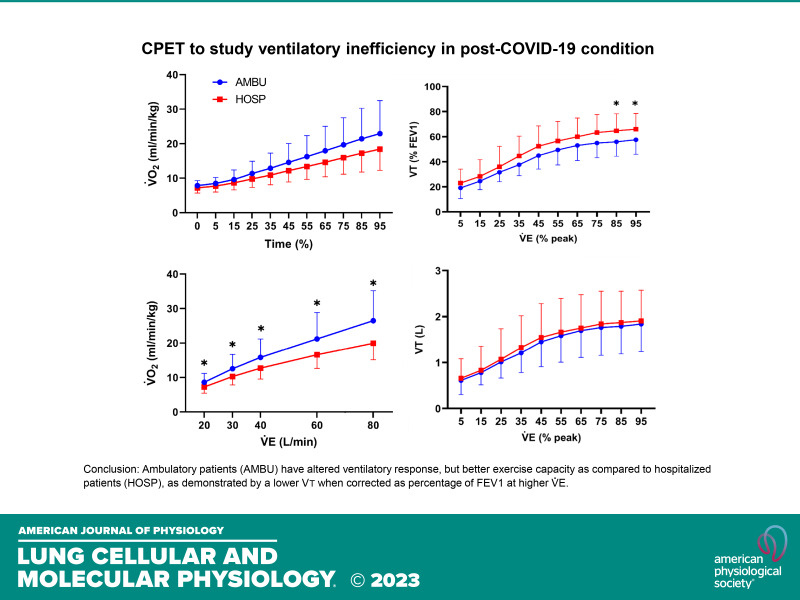

## INTRODUCTION

Infection with severe acute respiratory syndrome-coronavirus-2 (SARS-CoV-2) can result in a wide range of clinical presentations from asymptomatic to acute respiratory distress syndrome (ARDS) and eventually death (1). The term post-COVID-19 condition (PCC) has been defined as persistent respiratory symptoms 3 mo after SARS-CoV-2 infection (2–7). It is problematic as it is mainly based on symptoms for its diagnosis. Current hypotheses for PCC consider the combination of subclinical pulmonary involvement (8) and psychiatric comorbidities (9–11) including mechanisms related to stress and trauma or peripheral limitation (12, 13). However, the exact pathophysiology remains unexplained.

In this context, cardiopulmonary exercise testing (CPET) is an important tool for the diagnostic strategy of dyspnea since it allows a comprehensive assessment of pulmonary, cardiovascular, musculoskeletal, and neurological function (14). It has been demonstrated to be effective to explore PCC (15–17) and ventilatory inefficiency (16, 18–22). Among these, hyperventilation syndrome (HVS) is commonly found in post-COVID-19 patients, regardless the severity of the initial clinical presentation (2–4). HVS has been defined as a dysregulation of ventilation leading to hypocapnia, in the absence of somatic causes of hyperventilation (23). Mechanisms leading to the ventilatory evolution during exercise in PCC (17, 20, 24) need to be described. To the best of our knowledge, a direct comparison of the ventilatory responses to exercise after COVID-19 of different severity was never attempted. We hypothesized that PCC of mild SARS-CoV-2 infection without abnormalities in pulmonary function tests or imaging and managed through ambulatory care has to be distinguished from the remaining respiratory symptoms posthospitalization (25–28) which are a consequence of the viral infection and the ensuing inflammatory response with lung damage, fibrosis, and possibly postintensive care polyneuropathy (29, 30). The purpose of the study is to demonstrate that ambulatory patients (AMBU) have a different ventilatory response to exercise compared with hospitalized patients (HOSP) due to different pathophysiology of SARS-CoV-2 infection and severity. Such a study may better explain the pathophysiology of post-COVID-19 respiratory symptoms and therefore define different phenotypes of PCC. The aim of the present work is to explore and compare the exercise ventilatory response in hospitalized and ambulatory post-COVID-19 patients regarding the evolution of the ventilation during exercise.

## MATERIALS AND METHODS

### Design and Study Population

We retrospectively included patients aged 18 and older with a PCR-proven SARS-CoV-2 infection, who underwent a CPET at the Geneva University Hospitals, Switzerland, between July 2020 and May 2022 for investigation of persisting symptoms after COVID-19. Once they provided their written informed consent, their anthropometric data, clinical history, CPET, chest computed tomography (CT) scan, and pulmonary function tests (PFTs) were gathered. Patients were divided in two groups: *1*) those who had required hospitalization (HOSP) and *2*) those who did not require O_2_ therapy or hospitalization during COVID-19 (AMBU). The study was part of the COVISQAR study (NCT04881214) and approved by the Geneva ethical committee (No. 2020-01457).

### Cardiopulmonary Exercise Testing

Cardiopulmonary exercise testing (CPET) consisted of an incremental ramp test, on an electromagnetic cyclo-ergometer (Variobike 500, ergoline GmbH, Bitz, Germany) with a 12-channel electrocardiography unit (Cardiopart 12 Blue ECGpro, AMEDTEC Medizintechnik Aue GmbH, Aue, Germany) and pulse oximeter at the fingertip (WristOx2 3150, Nonin Medical Inc, Plymouth, MN). Respiratory gas and flows were continuously measured breath-by-breath at the mouth, using a metabolic cart (Ergostik, Geratherm Respiratory GmbH, Bad Kissingen, Germany) calibrated with a 3 L syringe and two gases of known concentration. Eighty-five microliters of capillary blood was sampled from the ear lobe, at rest, and near maximal exercise, for lactate concentration ([La]b) and capillary blood gas determination (PcO_2_ and $Pc{CO}_{2}$ for oxygen and carbon dioxide, respectively; ABL90 FLEX, Radiometer Medical ApS, Brønshøj, Denmark). Reference values for CPET were calculated based on Hansen and Wassermann (31, 32).

Respiratory flows and gas were analyzed to obtain breath-by-breath minute ventilation (V̇e), O_2_ uptake (V̇o_2_), CO_2_ production (V̇co_2_), tidal volume (VT), breathing frequency (BF), end tidal partial pressure of CO_2_ ($\text{P}{\text{ET}}_{{\text{CO}}_{2}}$), and respiratory exchange ratio (RER) by the inbuilt software of the metabolic cart (Blue Cherry, Geratherm Respiratory GmbH, Bad Kissingen, Germany). The ventilatory anaerobic threshold (VAT) was identified with the aid of the same software and corrected using the V-slope method. Ventilatory inefficiency was defined as a value over the following ranges *1*) ventilation as a function of CO_2_ production (V̇e/V̇co_2_) slope [19.6–30.4], *2*) V̇e/V̇co_2_ intercept [−1.5 to 6.3], *3*) V̇e-V̇co_2_ relationship (VeqCO_2_) at nadir [25–29] (22, 33, 34).

After a 2-min quiet rest period, patients performed at least 3 min of warm-up at an intensity comprised between 0 W and 50 W. The warm-up was followed by an incremental ramp test up to volitional exhaustion. Modified Borg scale (mBorg) [0–10] was used for subjective quantification of dyspnea (35). The increments were individually selected based on the individual fitness level to allow exhaustion in 8–12 min. The increments ranged from 7.5 W to 20 W/min. Absolute and relative contraindications and exercise termination for CPET were applied according to the ATS/ACCP statement on cardiopulmonary exercise testing (14).

### Pulmonary Function Tests

Pulmonary function tests (PFTs) were completed using a differential pressure flowmeter (Geratherm Respiratory GmbH, Bad Kissingen, Germany) calibrated with a 3 L syringe and performed according to the current international guidelines (36). References values were calculated based on the Global Lung Function Initiative reference equations for forced vital capacity (FVC), forced expiratory volume in one second (FEV1), their ratio, and single-breath carbon monoxide transfer capacity (DLCO). Results are reported as percent predicted value (PPV) and z-score (37, 38).

### Chest CT Scan

Chest CT scans were performed when post-COVID-19 lung parenchymal sequelae were suspected. The reading was performed by the radiologist (A.J.L.) and the two respiratory physicians (F.L. and Iv.G.) of the study, reaching a consensus on the interpretation.

### Questionnaires

The patients were asked to answer to six questionnaires routinely collected in our center: *1*) the hospital anxiety and depression scale (HADS), *2*) the short form health survey (SF-36), *3*) the Saint George’s respiratory questionnaire (SGRQ), *4*) the Dyspnea-12 questionnaire (D-12), *5*) the Nijmegen score (NJ), and *6*) the London Chest Activity of Daily Living scale (LCADL).

### Data Analysis

Breath-by-breath data during the ramp test were analyzed under MATLAB (version 2020 b, The MathWorks, Inc., Natick, MA) to obtain: *1*) peak values as the highest of 30 s mobile means, *2*) the nadir of EqCO_2_ by averaging the three lowest consecutive 30 s data points, and *3*) V̇e/V̇co_2_ slope and *y*-intercept by the MATLAB ROBUSTFIT function calculated over the linear component of V̇e versus V̇co_2_ up to VAT, when identified, or over the whole ramp-test in case of impossible determination of VAT (33). The evolution of ventilatory and gas exchange parameters throughout the ramp test were analyzed both as a function of time and as a function of V̇e. To take into account the different exercise duration between subjects, the exercise time was normalized to individual exercise duration, resulting in similar time points, i.e., isotimes ranging from 5% to 95% of total time duration, with 10% bins. In order not to be influenced by a possible bronchial obstruction V̇e was also corrected to FEV1.

### Statistical Analysis

Continuous data are presented as median and interquartile range (median, [IQR]). Comparisons between groups (AMBU vs. HOSP) were performed using Mann–Whitney *U* tests. Categorical data are presented as counts and percentages. For comparison between groups, Pearson’s χ^2^ test was used, and in cases where any count was equal or less than 5, Fisher’s exact test was applied. When data were analyzed as a function of time or V̇e, a two-way ANOVA for repeated measures was used to identify the effect of each group. A two-tailed *P* value < 0.05 was considered statistically significant. All statistical analyses were performed with Prism (V9, GraphPad, La Jolla, CA).

## RESULTS

A total of 79 consecutive patients (median age, 54 [44–60] years; female 57%) were recruited; of these, 42 were AMBU and 37 were HOSP. Table 1 shows the baseline demographic data of the studied population. Compared with the AMBU, HOSP patients were older (*P* < 0.05), with a higher body mass index (BMI; *P* < 0.001), and more cardiovascular comorbidities (*P* < 0.05), but with the same proportion of smokers (*P* = 0.30). One patient from the HOSP group was previously recorded with mild chronic obstructive lung disease (FEV1/FVC: −1.84 z-score, FEV1: 95% PPV). Both groups reported mainly dyspnea (73%), followed by fatigue (27%). Patients were hospitalized for a median of 20 [8–34] days and mainly for pneumonia (41%) or ARDS (30%). HOSP underwent PFTs, CPET, and CT scan significantly earlier than AMBU (*P* < 0.050, Table 2). Among HOSP, 12 (32%) patients had abnormal values for spirometry and 18 (51%) for DLCO with a median PPV of 75% [62–93] (*P* < 0.001).

**Table 1.** Baseline characteristics: anthropometric data, comorbidities, reasons for evaluation, and characteristics of hospitalization of all patients and, separately, of ambulatory and hospitalized patients

	All	Ambulatory Patients	Hospitalized Patients	*P* Value
Number (females)	79 (45 F)	42 (29 F)	37 (16 F)	0.0208
Age, yr	54 [44–60]	47 [38–55]	57 [51–61]	0.0026
Weight, kg	76 [69–89]	73 [64–80]	86 [72–99]	< 0.0001
Height, cm	170 [161–175]	168 [161–174]	171 [163–177]	0.3310
BMI, kg·m^−2^	27.8 [23.8–31.2]	25.0 [22.1–29.3]	29.8 [26.1–34.1]	0.0002
Comorbidities, *n* (%)				
Cardiovascular diseases	18 (23%)	5 (12%)	13 (35%)	0.0140
Systemic hypertension	14 (18%)	4 (10%)	10 (27%)	0.0313
Other cardiovascular diseases	6 (8%)	1 (2%)	5 (14%)	0.0659
Pulmonary diseases	20 (25%)	11 (26%)	9 (24%)	0.8490
Obstructive sleep apnoea	11 (14%)	5 (12%)	6 (16%)	0.5807
Asthma	9 (11%)	5 (12%)	4 (11%)	0.2730
Other pulmonary diseases	2 (3%)	1 (2%)	1 (3%)	0.5044
Endocrine diseases	31 (39%)	8 (19%)	23 (62%)	< 0.0001
Obesity	26 (33%)	8 (19%)	18 (49%)	0.0052
Other endocrine diseases	7 (9%)	0	7 (19%)	
Current or previous neoplasia	8 (10%)	5 (12%)	3 (8%)	0.2534
Other diseases	12 (15%)	5 (12%)	7 (19%)	0.3861
No comorbidities	18 (23%)	13 (31%)	5 (14%)	0.0652
Current or previous tobacco smoke	7 (9%)	4 (10%)	3 (8%)	0.3000
Reasons for evaluation, *n* (%)				
Dyspnoea	58 (73%)	36 (86%)	22 (59%)	0.0084
Fatigue	21 (27%)	16 (38%)	5 (14%)	0.0136
Memory and/or concentration disorders	14 (18%)	10 (24%)	4 (11%)	0.0781
Cough	12 (15%)	7 (17%)	5 (14%)	0.6968
Palpitations	8 (10%)	5 (12%)	3 (8%)	0.2534
Sleep disorders	8 (10%)	7 (17%)	1 (3%)	0.0383
Muscular pain	7 (9%)	5 (12%)	2 (5%)	0.1954
Chest pain or discomfort	6 (8%)	3 (7%)	3 (8%)	0.3209
Headache	5 (6%)	5 (12%)	0	
Other symptoms	10 (13%)	8 (19%)	2 (5%)	
Hospitalization				
Duration, days			20 [8–34]	
Reason for hospital admission, *n* (%)				
Pneumonia			24 (65%)	
ARDS			13 (35%)	
Pulmonary embolism			8 (22%)	

Continuous data are reported as median [interquartile range] and the corresponding *P* value refers to Mann–Whitney test result; categorical data are reported as counts (*n*) and percentage proportion with respect to the corresponding total number (%). The related *P* value refers to the Pearson’s χ^2^ or Fisher’s exact test. ARDS, acute respiratory distress syndrome; BMI, body mass index; F, female.

**Table 2.** Results from the pulmonary function tests, the cardiopulmonary exercise testing, and of the chest CT scan of all patients and, separately, of ambulatory and hospitalized patients

	All	Ambulatory Patients	Hospitalized Patients	*P* Value
Pulmonary function tests				
Number	79	42	37	
Days after COVID-19	179 [124–342]	273 [142–376]	146 [112–183]	0.0050
FVC (% predicted)	93 [84–104]	97 [86–108]	90 [78–99]	0.0101
FVC z-score < −1.64 [*n* (%)]	10 (13%)	1 (2%)	9 (24%)	0.0036
FEV1 (% predicted)	95 [89–104]	98 [90–110]	94 [83–101]	0.0452
FEV1 z-score < −1.64 [*n* (%)]	9 (11%)	1 (2%)	8 (22%)	0.0079
FEV1/FVC (% of predicted)	103 [97–107]	100 [96–105]	105 [100–111]	0.0113
FEV1/FVC z-score < −1.64 [*n* (%)]	2 (3%)	0	2 (5%)	0.2162
Number	71	36	35	
Days after COVID-19	152 [110–226]	204 [134–349]	131 [107–157]	0.0028
DLCO (% predicted)	84 [70–97]	91 [80–100]	75 [62–93]	0.0031
DLCO z-score < −1.64 [*n* (%)]	22 (31%)	4 (11%)	18 (51%)	0.0002
KCO (% predicted)	95 [83–106]	95 [84–104]	95 [81–108]	0.9506
KCO z-score < −1.64 [*n* (%)]	9 (13%)	5 (14%)	4 (11%)	0.2650
CPET				
Number	79	42	37	
Days after COVID-19	194 [135–396]	311 [57–440]	159 [127–193]	0.0005
Values at rest				
RER	0.84 [0.78–0.94]	0.87 [0.79–0.98]	0.82 [0.78–0.9]	0.1615
$\text{P}{\text{ET}}_{{\text{CO}}_{2}}$, mmHg	32 [29–34]	32 [27–34]	32 [31–34]	0.3148
$Pc{CO}_{2}$, kPa	4.8 [4.4–5.0]	4.9 [4.4–5.0]	4.6 [4.3–5.0]	0.5454
$P\text{A}-a_{O_{2}}$, mmHg	19.2 [12.2–28.0]	15.8 [10.7–23.5]	22.1 [17.0–31.8]	0.0038
Values at exercise peak				
Power (W)	129 [100–179]	126 [74–180]	147 [97–178]	0.9591
Power (% of predicted)	93 [74–109]	98 [83–123]	90 [71–103]	0.0672
Heart rate, min^−1^	159 [140–171]	160 [143–180]	155 [134–167]	0.1100
Heart rate (% of predicted)	94 [87–99]	93 [87–98]	94 [84–99]	0.9279
Heart rate > 90% [*n* (%)]	50 (63%)	27 (64%)	23 (62%)	0.8451
[La]b, mmol·L^−1^	9.2 [7.1–11.7]	9.7 [7.1–11.9]	9.2 [7.1–11.9]	0.9136
V̇o_2_, L·min^−1^	1.59 [1.22–2.11]	1.54 [1.22–2.08]	1.66 [1.34–2.14]	0.6513
V̇o_2_, mL·min^−1^·kg^−1^	19.6 [16.1–23.3]	20.9 [16.7–29.7]	19.0 [14.1–20.1]	0.0319
V̇o_2_ (% of predicted)	81 [74–100]	86 [77–101]	80 [71–99]	0.4012
Ventilation, L·min^−1^	79 [62–105]	78 [62–98]	82 [62–115]	0.5047
Tidal volume, L	1.91 [1.47–2.37]	1.97 [1.51–2.36]	1.97 [1.40–2.50]	0.8816
PcO_2_, kPa	11.5 [10.4–12.8]	12.0 [11.1–13.3]	10.9 [10.1–12.0]	0.0026
$P\text{A}-a_{O_{2}}$, mmHg	28.0 [17.3–37.6]	21.0 [14.0–31.1]	35.3 [24.1–40.5]	0.0002
VD/VT ratio	26.6 [19.6–35.7]	25.6 [18.7–33.7]	27.0 [20.7–36.4]	0.3451
Breathing frequency, min^−1^	44 [39–50]	44 [39–50]	47 [40–50]	0.6439
Borg	7 [5–9]	8 [5–9]	7 [5–9]	0.4749
$Sp_{O_{2}}$ (%)	96 [94–97]	97 [95–98]	94 [92–96]	0.0002
Derived measurements				
Delta $\text{P}{\text{ET}}_{{\text{CO}}_{2}}$	−2 [-4 to 1]	−2 [−3 to 1]	−1 [−4 to 1]	0.8857
V̇e/V̇co_2_ slope	30.9 [26.1–34.3]	30.3 [25.2–36.3]	31.0 [28.2–33.5]	0.9630
V̇e/V̇co_2_ y-intercept, L·min^−1^	4.2 [2.7–6.2]	4.1 [2.5–6.7]	4.4 [2.7–5.7]	0.9164
VeqCO_2_ at nadir	32.5 [28.4–36.0]	32.2 [27.6–37.5]	33.2 [29.3–35.4]	0.7361
Chest CT scan				
Number	59	26	33	
Days after COVID-19	445 [137–585]	580 [495–625]	147 [122–325]	< 0.0001
Abnormal [*n* (% of total)]	39 (66%)	11 (44%)	28 (85%)	0.0006
GGO	20 (34%)	2 (8%)	18 (55%)	0.0001
Consolidation	3 (5%)	0	3 (9%)	0.1678
Bands	16 (27%)	2 (8%)	14 (42%)	0.0024
Reticulation	12 (20%)	1 (4%)	11 (33%)	0.0045
Trapped air	13 (22%)	1 (4%)	12 (36%)	0.0023
Honeycomb	2 (3%)	1 (4%)	1 (3%)	0.5015
Bronchiectasis	5 (8%)	2 (9%)	3 (8%)	0.3542
Emphysema	2 (3%)	1 (4%)	1 (3%)	0.5015
Atelectasis	7 (12%)	2 (8%)	5 (15%)	0.2261
Mosaïc pattern (MinIP)	9 (15%)	1 (4%)	8 (24%)	0.0287
Pulmonary embolism	2 (3%)	2 (8%)	0	0.1899
Abnormal perfusion	10 (17%)	2 (8%)	8 (24%)	0.0718

Continuous data are reported as median [interquartile range] and the corresponding *P* value refers to Mann–Whitney test result. Categorical data are reported as counts (*n*) and percentage proportion with respect to the corresponding total number (%), the related *P* value refers to the Pearson’s χ^2^ or Fisher exact test. CPET, cardiopulmonary exercise testing; DLCO, carbon monoxide transfer capacity; FEV1, forced expiratory volume in 1 s; FVC, forced vital capacity; GGO, ground-glass opacity; KCO, CO transfer coefficient; [La]b, blood lactate concentration; MinIP, minimum intensity projection; $P\text{A}-a_{O_{2}}$, alveolar-arterial Po_2_ difference; Pc, capillary partial pressure; RER, respiratory exchange ratio; $Sp_{O_{2}}$, peripheral O_2_ saturation; VD/VT, dead volume – tidal volume ratio; V̇e/V̇co_2_, ventilation as a function of CO_2_ production; VeqCO_2_, V̇e-V̇co_2_ relationship; V̇o_2_, oxygen consumption.

Regarding CPET, we observed a significant difference in peak V̇o_2_ when normalized per body mass (*P* = 0.03, Table 2), which is consistent with the reported difference of BMI between the two groups. All tests reached objective maximal criteria following the ATS/ERS criteria (14) excepted for two that were conducted to maximal subjective exhaustion (mBorg > 8). Before CPET, resting $Pc{CO}_{2}$ was 4.9 [4.4–5.0] kPa in AMBU and 4.6 [4.3–5.0] kPa in HOSP (*P* = 0.36). $\text{P}{\text{ET}}_{{\text{CO}}_{2}}$ decreased slightly during exercise, i.e., −2 [−3 to 1] in AMBU and −1[−4 to 1] in HOSP. CPET exercise duration was 918 [805–1,037] s in AMBU and 880 [764–1,087] s in HOSP (*P* = 0.69). Peak CPET exercise values were not significantly different in terms of power (*P* = 0.96), heart rate (*P* = 0.11), lactates (*P* = 0.91), nor absolute V̇o_2_ (*P* = 0.65; Table 2). mBorg scale at the end of the exercise was 7 [5–9] for HOSP and 8 [5–9] for AMBU, with no difference between groups (*P* = 0.47). V̇e/V̇co_2_ slope (30.3 [26.1–34.3] in AMBU vs. 31.0 [28.2–33.5] in HOSP, *P* = 0.96) and EqCO_2_ at nadir (32.2 [27.6–37.4] in AMBU vs. 33.2 [29.3–35.4] in HOSP, *P* = 0.74) were elevated (33) in both groups. Nonetheless, peak breathing frequency was within normal range (44 [39–50] in AMBU and 47 [40–50] in HOSP, *P* = 0.64) and peak VD/VT was not increased (25.6 [18.7–33.7] in AMBU vs. 27.0 [20.7–36.4] in HOSP, *P* = 0.35).

Regarding chest CT scan, 2 AMBU showed ground glass opacities whereas 28 of HOSP had abnormalities of which 55% were ground-glass abnormalities, 42% bands, and 33% reticulations. None had an element in favor of pulmonary thrombo-embolic disease (Table 2).

There were no significant differences in questionnaire results (SF-36, SGRQ, D-12, LCADL, NJ, HADS) between the two groups despite a trend to worse physical impact in HOSP group. Twenty-four percent had a pathological value of the HADS score with the same proportion in the evaluation of depression and anxiety (Supplemental Table S1). Half of our patients had a positive NJ (> 23) whereas 24% showed an elevated HADS. There was no association between these two scores and ventilatory inefficiency (Supplemental Table S2). We found a moderate correlation between HADS and NJ (*r* = 0.56, *P* < 0.001 for HADS-Anxiety score, *r* = 0.54, *P* < 0.001 for HADS-Depression score). The NJ score also had a trend to higher scoring in AMBU compared with HOSP without reaching a significant difference (respectively, 24 [15–32] vs. 19 [11–27], *P* = 0.21).

**Figure 1.**
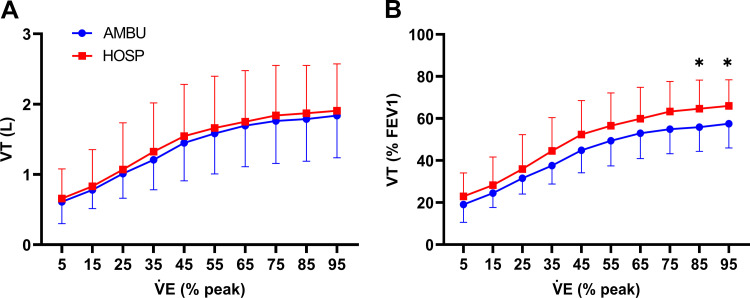
Tidal volume (VT) as a function of increasing ventilation (V̇e) expressed as percentage of its peak value. *A*: VT expressed as absolute value. *B*: VT expressed in percentage of forced expiratory volume in 1 s (FEV1). Comparison between ambulatory (AMBU, blue line and dots) and hospitalized (HOSP, red line and squares) patients. *P < 0.05 between groups.

**Figure 2.**
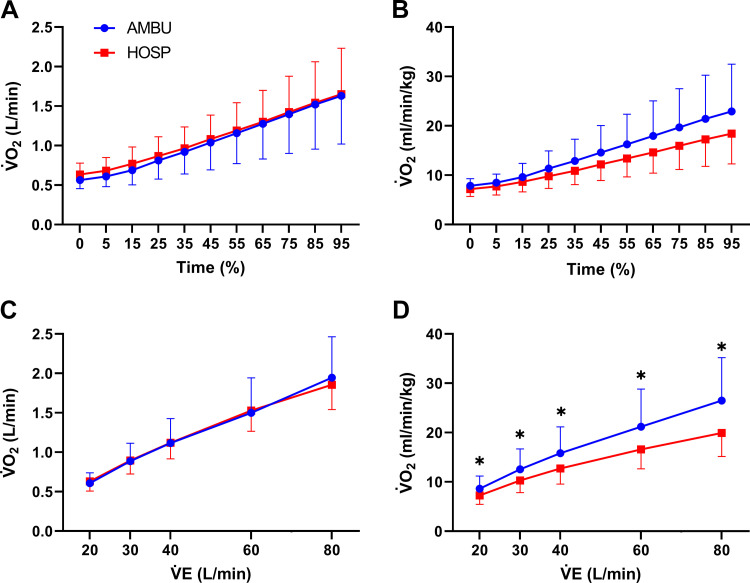
Comparison between ambulatory (AMBU; blue line and dots) and hospitalized (HOSP; red line and squares) patients. *A*: oxygen consumption (V̇o_2_) as a function of time of exercise (expressed in percentage of total time of cardiopulmonary exercise testing). *B*: V̇o_2_ as a function of V̇e at several timepoints. *C* and *D*: V̇o_2_ expression relative to body weight. *P < 0.05 between groups.

Figure 1*B* illustrates that AMBU had a lower VT/(%FEV1) during exercise with respect to HOSP (ANOVA *P* = 0.0088). Figure 2 shows the relationship between V̇o_2_ absolute and relative to body weight and time of exercise or V̇e. A group effect was identified when relative V̇o_2_ was plotted against time of exercise (Fig. 2*B*, ANOVA *P* = 0.016), and AMBU showed a higher relative V̇o_2_ with respect to HOSP at V̇e ranging from 20 to 80 L·min^−1^ (Fig. 2*D*, ANOVA *P* < 0.001). This difference was not significant when plotted against absolute V̇o_2_ (Fig. 2, *A* and *C*).

## DISCUSSION

In this observational study we compared the exercise ventilatory response between AMBU and HOSP patients with persisting respiratory symptoms after SARS-CoV-2 infection. We found that the severity of COVID-19 influences the exercise ventilatory response but not the exercise capacity. Both AMBU and HOSP had a similar and preserved peak power with AMBU showing altered ventilatory response to exercise.

When analyzing the peak exercise values, both groups showed similar, preserved metabolic and mechanical power. Despite having normal exercise capacity, both groups exhibited an impaired ventilatory adaptation to exercise. All patients showed an increased V̇e/V̇co_2_ slope, a $Pc{\mathrm{CO}}_{2}$ at the lower limit of normal at rest, as well as a small $\text{P}{\text{ET}}_{{\text{CO}}_{2}}$ decrease during exercise. Such ventilatory response might be explained in the HOSP group by the well-known deconditioning (12, 16) and pulmonary sequelae (13, 19) following a hospitalization for COVID-19 whereas a different origin should be hypothesized for AMBU. Notably, AMBU had a greater alteration of the ventilatory response than HOSP despite the former being younger, with a lower BMI, and without evidence of lung damage. Our results showed that AMBU had a less efficient ventilation than HOSP, as demonstrated by a lower VT relative to FEV1 at high V̇e. The absence of difference between groups in V̇e/V̇co_2_ slope illustrates that thorough analysis of each component of the ventilation (meaning VT and BF) may help reveal otherwise unidentified ventilator inefficiency. Thus, underling the role of ventilatory inefficiency in the development of dyspnea. Several authors suggest to use normalization of VT to allow intersubject comparison and avoid confounding effects of height or any alterations in lung volume or flow (24, 39–41). Typically, they use FVC or resting inspiratory capacity. We chose to adjust VT for FEV1 to reduce the potential bias induced by any eventual obstructive and restrictive disease and to account for significant differences regarding FEV1 between the two groups.

Among the abnormal ventilatory responses in PCC, the HVS has been widely described (3, 4, 20). Although it has no gold standard diagnostic criteria, HVS relies on the exclusion of organic pathology and the use of several methods of assessment (42). During a CPET, a low arterial blood CO_2_ pressure ($Pa_{{CO}_{2}}$), $\text{P}{\text{ET}}_{{\text{CO}}_{2}}$ at rest, high V̇e/V̇co_2_ slope, small $\text{P}{\text{ET}}_{{\text{CO}}_{2}}$ decrease, and high BF may suggest an HVS (22, 43). Our patients showed some characteristics of HVS except for a high BF and lower limits of normal for $Pc{CO}_{2}$, and $\text{P}{\text{ET}}_{{\text{CO}}_{2}}$ at rest, and a normal VD/VT. We can thus hypothesize that post-COVID-19 patients may suffer from a more complex ventilatory pattern disorder than a mere HVS (20, 21). From a pathophysiological point of view, an increase in central neural respiratory drive has been proposed to be associated to HVS in post-COVID-19 dyspnea (4) in the context of dysautonomia. This may imply a disruption of the ventilatory control through stimulation of activating systems, suppression of inhibitory systems, or stimulation of intrathoracic receptors (44). The hypothesis under the respiratory physiopathology may be similar to the case of patients with cardiac dysautonomia (45, 46), which have been described to have higher V̇e/V̇co_2_ slope and BF.

Investigation of the impact of COVID-19 on the quality of life (QoL), dyspnea, and perceived exercise capacity displayed no differences between AMBU and HOSP (Supplemental Table S1). With respect to previous literature, our results demonstrated a better recovery than that observed in other post-viral infections (47), but still reflect a real impact on QoL as compared with control healthy population (48). It is remarkable that the impact on QoL was of a comparable severity between HOSP and AMBU patients. One may suggest that a mild disease did not protect against a significant influence on QoL. In addition, it is possible that HOSP patients might have been more effectively protected against worsening of QoL due to the care they received during hospitalization (11). HADS and NJ did not correlate to abnormal ventilatory response (Supplemental Table S2) but shown a good in-between association. This may reflect the lack of discriminant validity for physical distress of HADS and the influence of depression and anxiety on the NJ. The prevalence of elevated NJ scores in both groups confirms a lower accuracy of the questionnaire, particularly in the context of anxiety and in the presence of a more complex ventilatory pattern than just HVS as observed in PCC. Although HOSP were characterized by a more severe form of COVID-19, pulmonary pathological findings, and were tested earlier after the infection, AMBU QoL was still similarly affected months after the infection. This underlies the importance of a comprehensive management of patients suffering from persistent respiratory symptoms after a mild form of COVID-19.

Overall, this study demonstrates that the analysis of the ventilatory response to exercise may unveil pathological findings in subjects suffering from persistent symptoms after a mild form of COVID-19. In fact, this population may be characterized by normal PFTs and CT scan findings as well as a preserved exercise capacity measured at CPET. The pathophysiology of their persistent symptoms would remain unexplained without further investigations. Aparisi et al. (18) found a greater ventilatory inefficiency with a V̇e/V̇co_2_ slope of 33.1 [30.9–38.8] in hospitalized patients compared with 27.6 [23.6–29.8] in ambulatory patients. These authors analyzed patients with and without dyspnea. We propose that the criteria for the diagnosis of HVS should be inspected and the analysis of the VT-V̇e relationship should be performed to identify a possible alteration of the ventilatory response to exercise that might explain the patient’s symptoms. Of note, the VT-V̇e relationship should be performed also by normalizing the VT for the subject’s FEV1 to diminish the confounding effect of the body size and, possibly, of pulmonary obstructive pattern. As proposed in our study, the analysis of the VT-V̇e relationship at several isotimes may be more sensitive to unveil ventilatory abnormalities. The evolution of the V̇e/VT ratio has never been included in the definition of ventilatory inefficiency, but the evolution of VT during exercise appears to be of paramount importance. Indeed, it represents the main component of the V̇e increase during the initial part of exercise. Unfortunately, the small sample size of our investigated population prevented us to perform a subgroup analysis of our population, e.g., by separating HOSP in patients who were hospitalized in the intensive care unit and the general ward.

Another limitation of our studies is related to the heterogeneity in the distribution of comorbidities between the HOSP and AMBU groups. Hence, it is well described that heart failure may participate to impairment of ventilator efficiency (49) and may have influenced our results. However, the main cardiovascular comorbidity whose prevalence was significantly different between the two groups was systemic hypertension, and patients with heart failure or severe other cardio-respiratory diseases were excluded from the study. Therefore, it is unlikely that the results of the present work would have been significantly influenced by such conditions.

### Conclusions

The severity of COVID-19 influences the ventilatory response, but not the exercise capacity in patients with persistent symptoms. Despite similar V̇e/V̇co_2_ slope, V̇e/V̇co_2_ intercept, V̇e-V̇co_2_ relationship at nadir, we found lower VT relative to FEV1 at higher V̇e in AMBU patients compared with HOSP patients. Further investigations on the ventilatory response to exercise are needed to improve our understanding of the pathophysiology of dyspnea and phenotyping of PCC patients.

## DATA AVAILABILITY

The data that support the findings of this study are available from the corresponding author upon reasonable request.

## SUPPLEMENTAL DATA

10.6084/m9.figshare.23802900 Supplemental Table S1: https://doi.org/10.6084/m9.figshare.23802900.

10.6084/m9.figshare.23802900 Supplemental Table S2: https://doi.org/10.6084/m9.figshare.23802900.

## GRANTS

This study was supported by an unrestricted grant from the Ligue Pulmonaire Genevoise to the COVISQAR study (No. NCT04881214).

## DISCLOSURES

F.L. reports personal fees from MSD and Janssen, contracted as consultant, and participate to steering committee for MSD, Bayer and Janssen, outside the submitted work. None of the other authors has any conflicts of interest, financial or otherwise, to disclose.

## AUTHOR CONTRIBUTIONS

Iv.G. and F.L. conceived and designed research; Iv.G., A.B. and A.J.D.S.-L.-L. performed experiments; Iv.G., A.B., and A.T. analyzed data; Iv.G., A.B., L.B., A.J.D.S.-L.-L., A.T., and F.L. interpreted results of experiments; Iv.G., A.B., and A.T. prepared figures; Iv.G. and F.L. drafted manuscript; Iv.G., A.B., A.T., and F.L. edited and revised manuscript; Iv.G., A.B., M.N., I.G., L.B., A.J.D.S.-L.-L., A.T., and F.L. approved final version of manuscript.
